# Vivaldi: Visualization and validation of biomacromolecular NMR structures from the PDB

**DOI:** 10.1002/prot.24213

**Published:** 2012-11-23

**Authors:** Pieter M S Hendrickx, Aleksandras Gutmanas, Gerard J Kleywegt

**Affiliations:** Protein Data Bank in Europe, EMBL-European Bioinformatics Institute, Wellcome Trust Genome CampusHinxton, Cambridge CB10 1SD, United Kingdom

**Keywords:** Protein Data Bank, nuclear magnetic resonance, chemical shifts, distance constraints, angular constraints, residual dipolar couplings

## Abstract

We describe Vivaldi (VIsualization and VALidation DIsplay; http://pdbe.org/vivaldi), a web-based service for the analysis, visualization, and validation of NMR structures in the Protein Data Bank (PDB). Vivaldi provides access to model coordinates and several types of experimental NMR data using interactive visualization tools, augmented with structural annotations and model-validation information. The service presents information about the modeled NMR ensemble, validation of experimental chemical shifts, residual dipolar couplings, distance and dihedral angle constraints, as well as validation scores based on empirical knowledge and databases. Vivaldi was designed for both expert NMR spectroscopists and casual non-expert users who wish to obtain a better grasp of the information content and quality of NMR structures in the public archive. © Proteins 2013. © 2012 Wiley Periodicals, Inc.

## INTRODUCTION

The Protein Data Bank (PDB)^1^,^2^ archive is a rich repository of data and information on the structure and function of biologically relevant macromolecules and their complexes. The archive currently contains over 84,500 entries (referencing over 28,000 unique UniProt^3^ accession codes), of which almost 10,000 NMR-derived structures (almost 5000 unique UniProt codes, Table I). The PDB archive is managed by the Worldwide Protein Data Bank organization (wwPDB),^4^ which consists of the Research Collaboratory for Structural Bioinformatics (RCSB)^5^ and the BioMagResBank (BMRB)^6^ in the USA, the Protein Data Bank Japan (PDBj),^7^ and the Protein Data Bank in Europe (PDBe; http://pdbe.org/).^8^ Atomic models deposited to the PDB are curated by RCSB, PDBe, and PDBj, while experimental NMR data are processed by BMRB. As one of the founding wwPDB partners, PDBe provides annotated data on three-dimensional (3D) structures of biomacromolecules to the scientific community as well as advanced services based on these structures.

**Table I:** Data Coverage

	Source	PDB archive	NMR entries
Total number of entries	PDB^1^,^2^	84,508	9616
Unique UniProt^3^ accession codes referenced	SIFTS^49^	28,104	4822
Pfam^50^ sequence families referenced	SIFTS	6143	2455
CATH^51^ domain architectures present	SIFTS	2549	660
SCOP^52^ domain architectures present	SIFTS	4191	1150
Cluster analysis	OLDERADO^14^	−	7289
Chemical shift analysis	VASCO^28^	−	3361
Distance constraints	BMRB-NRG^36^	−	5978
Dihedral constraints	BMRB-NRG	−	3850
RDCs	BMRB-NRG	−	602
Validation scores	NRG-CING^47^	−	9491

Coverage of the protein universe in the PDB, and data available in Vivaldi for NMR entries (as of September 12, 2012). Up to date data coverage statistics can be found at http://pdbe.org/nmrstats/.

As the PDB archive continues to grow, the provision of adequate validation tools to its users becomes increasingly important.^9^ In many cases, the archive contains multiple structures of the same or similar molecules, for example, solved by competing groups or by different experimental techniques, with different ligands or containing mutations. Choosing the most appropriate structure in such cases is not an easy task for experts, let alone for non-specialist users. Even if only a single structure is known of a molecule of interest, it is still essential to assess whether the structure is suitable for the intended use.

**Figure 1:**
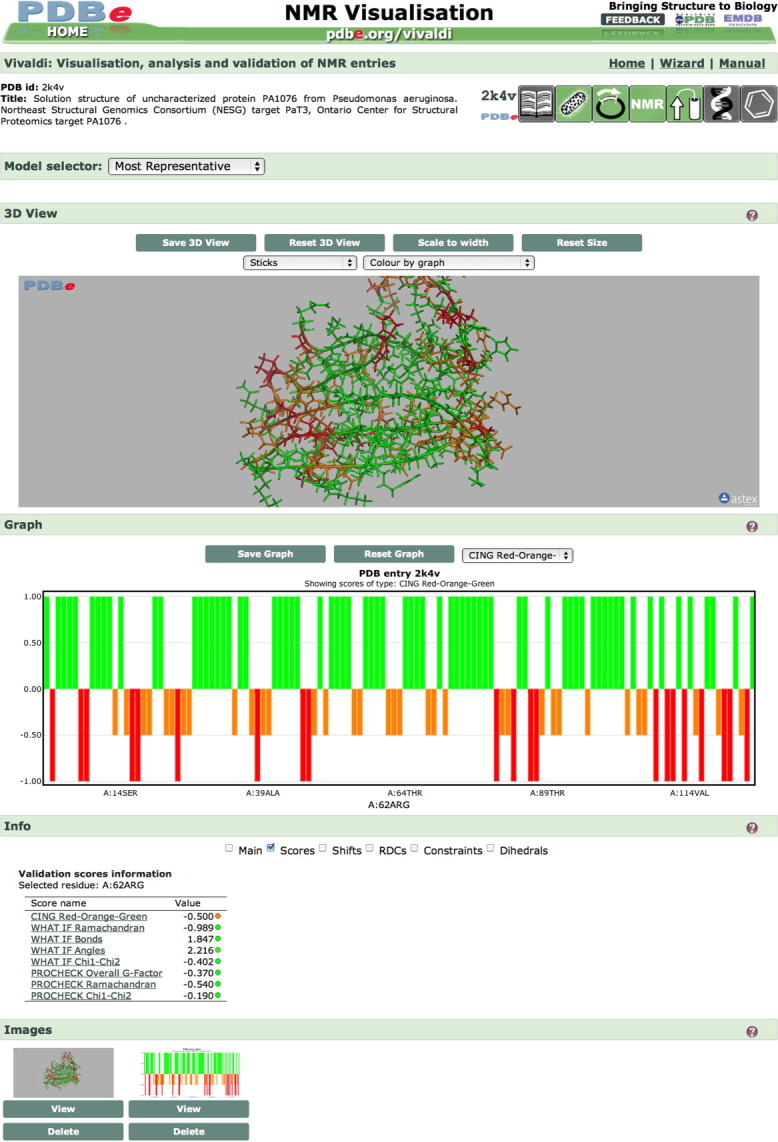
Layout of a Vivaldi page showing the default view for protein PA1076 from *Pseudomonas aeruginosa* (PDB entry 2k4v).^15^ The page contains a header including PDBprints,^9^ an interactive 3D viewer (OpenAstexViewer),^48^ a graph and a textual information section. The most representative model according to OLDERADO^14^ cluster analysis is displayed in the 3D viewer and the graph shows the NRG-CING^47^ red-orange-green (ROG) scores for this protein.

A number of validation software packages are available,^10^–^12^ but a single robust and widely-accepted validation score or standard set of validation criteria is yet to be defined for NMR-derived biomacromolecular structures. Therefore, a wwPDB NMR validation task force (VTF) has been convened to define standard validation criteria that will be applied by all wwPDB partners to all depositions of NMR structures (http://www.wwpdb.org/workshop/2010/nmr_validation.html), in analogy with the wwPDB X-ray VTF.^13^ In an effort to make information about the quality of NMR structures in the PDB accessible to the wider scientific community, PDBe has developed an interactive web-based tool called Vivaldi (Visualization and Validation Display; http://pdbe.org/vivaldi; Fig.1).

## MATERIALS AND METHODS

Several types of information are accessible through the Vivaldi service, including information about the homogeneity of the modeled structure ensemble, validation of the available experimental NMR data, and knowledge-based validation of the modeled conformations. The decisions on which sources of data and which validation criteria to include in Vivaldi were based on three factors: (a) accessibility, (b) the sources being well-established and published, and (c) data coverage of the PDB archive. In the future, Vivaldi will be modified to accommodate the recommendations of the wwPDB NMR VTF.

Typically, an NMR entry in the PDB archive contains 20 models of a macromolecule or a complex. For all intents and purposes, these are deposited in an arbitrary order and therefore no single model can be assumed to be more important than the others without analyzing the structure ensemble in more detail. One such type of analysis is performed by OLDERADO,^14^ which is used by PDBe to determine core domains within proteins and to cluster together individual models of the ensemble. OLDERADO identifies the most representative model of each cluster and the most representative model of the entire ensemble. With the Vivaldi web service, users can interactively explore the results of this cluster and domain analysis, overlay different models and visualize similarities and differences within and between clusters. Furthermore, the most representative model as identified by OLDERADO is used throughout Vivaldi as the one displayed by default (rather than, for instance, the first model of the ensemble). Supporting Information Figure S1(a) shows representative cluster models as identified by OLDERADO for PDB entry 2k4v,^15^ while Supporting Information Figure S1(b) highlights the domains on the most representative model of the ensemble.

To help users assess the local variability in the deposited ensemble of structures, Vivaldi calculates a simple dihedral order parameter,^16^ *S*^2^, for each residue in protein and nucleic acid molecules [Eq.(1)] and presents this information as a graph of *S*^2^ vs. residue number [Supporting Information Fig. S1(c)]. Selecting a residue in the graph displays a table with the dihedral angle values, pie-charts for the relevant dihedral angles and, if appropriate, a Ramachandran plot [Supporting Information Fig. S1(d)].


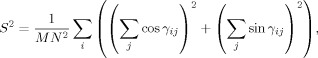
(1)

where γ are backbone dihedral angles (φ and ψ for proteins and α–ζ for nucleic acids), index *i* runs over the *M* different dihedral angles and index *j* runs over the *N* conformers of the ensemble. Values of *S*^2^ close to 1 indicate that the dihedral angles show little variation across the ensemble.

Chemical shifts are arguably the most studied NMR parameters with respect to the covalent structure, local conformations, and immediate spatial surroundings. The complex dependence of the chemical shift on an atom's chemical environment has resulted in a wide variety of shift prediction and validation tools based on different approaches (e.g., quantum-chemical calculations and neural networks) and taking into account different aspects of the chemical environment (e.g., solvent effects, aromatic ring currents, and hydrogen bonds). Some of the more recent software packages include AVS,^17^ CamShift,^18^ LACS,^19^,^20^ TALOS+,^21^ ShiftX2,^22^,^23^ SPARTA+,^24^,^25^ CheShift,^26^ DANGLE,^27^ and VASCO.^28^ As deposition of chemical shift information has been mandatory for NMR structures since December 2010, chemical shifts are becoming an increasingly important source of data for validating PDB structures.

For Vivaldi, VASCO^28^ validates experimental chemical shifts of proteins in a two-step process. First, the optimal referencing offset for each nucleus type (^1^H, ^15^N, ^13^C_aliphatic_, ^13^C_aromatic_, and ^13^C′) is calculated by comparing the reported chemical shifts to a set of protein chemical shifts that were correctly referenced by hand. Second, for each atom a database of chemical shifts is queried to identify atoms in a similar environment, defined as a combination of atom and residue type, secondary structure, and solvent accessibility. If a sufficient number of similar atoms are found, the *Z*-score of the reported chemical shift is calculated and if the absolute *Z*-score exceeds a value of 3 (99.7% confidence interval assuming a true normal distribution) the shift is labeled as an outlier.

**Figure 2:**
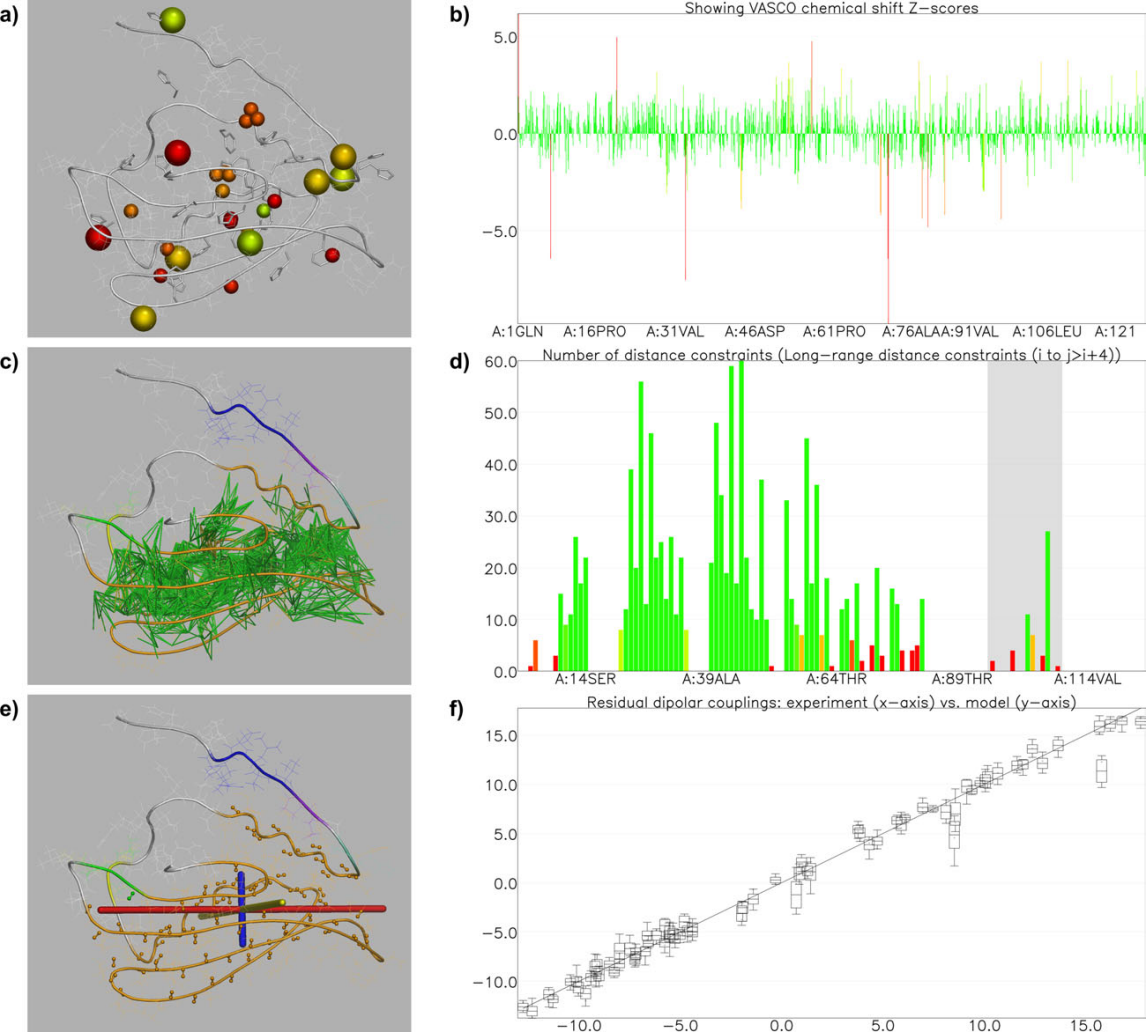
Vivaldi visualization of experimental data for protein PA1076 from *Pseudomonas aeruginosa* (PDB entry 2k4v).^15^ (**a**,**b**) Presentation of VASCO^28^ analysis of chemical shifts. (a) Nuclei with unusual chemical shifts are shown as spheres in the 3D viewer; aromatic residues are shown as sticks, to remind the user that the effect of aromatic ring currents is not explicitly accounted for in the VASCO analysis. (b) The same information is displayed in an interactive graph vs. residue number. (**c**,**d**) Analysis of distance constraints. (c) The most representative model of the ensemble is shown, colored by rigid-body domains as determined by OLDERADO.^14^ All long-range distance constraints (five or more residues apart in sequence) are shown as green sticks in the 3D viewer. (d) Graph of the number of long-range distance constraints per residue. The shaded area of the graph corresponds to helix 91–105 (UniProt numbering). (**e**) The chemical groups, for example N–HN, for which RDCs are available are shown as balls and sticks. Principal axes of the alignment tensor are also displayed. (**f**) Correlation plot of calculated vs. experimental RDCs. For each datapoint, the middle bar shows the calculated RDC averaged over the ensemble, and the box represents the standard deviation, while the whiskers are the minimum and maximum values calculated from the ensemble.

Vivaldi shows these outliers as solid spheres in its 3D viewer and as bars in the interactive graph [Fig.2(a,b)] with default coloring for both the spheres and the bars varying smoothly from green (*Z*-score < 2) via yellow (*Z*-score = 3.5) to red (*Z*-score > 5) depending on the degree of the deviation from the expected range. Aromatic ring currents, which can have a profound influence on chemical shifts, are not explicitly taken into account by VASCO. Therefore, aromatic rings in the structure are highlighted in the 3D viewer as stick models. Furthermore, the user can select a residue by clicking on a bar in the graph or on an atom in the 3D viewer and thereby obtain a table of the original chemical shift values and the VASCO scores for the nuclei in that residue.

Distance and dihedral angle constraints, together with residual dipolar couplings (RDCs) constitute the bulk of the experimental data used during structure calculation for the majority of NMR-derived macromolecular structures in the PDB. Although these types of experimental data are used directly for structure calculation (hence, the models are expected to fit them rather well), validation can still pinpoint problems. The Queen^29^ and PSVS^10^ validation suites provide analyses of distance constraints, whereas PALES,^30^ REDCAT,^31^ and MODULE2^32^ can be used to analyze RDC data.

Vivaldi retrieves pre-processed CCPN projects,^33^ which include all the constraints, from the Database Of Converted Restraints (DOCR),^34^ which is part of the NMR Restraints Grid (NRG)^35^,^36^ at BMRB. Distance constraints are further processed to classify them into groups, that is, intra-residual, short (1 or 2 residues apart in sequence), medium (3 or 4 residues apart), and long-range (5 or more residues apart), as well as to identify constraint violations for each group. Vivaldi can show violated and satisfied constraints, graphically in charts and in the 3D viewer as well as in tables [Fig.2(c,d)]. Any individual constraint can be selected to show the atoms involved, the actual inter-atomic distance(s) from the displayed model(s) and the constraint's lower and upper limit. Weighted summations (*d*^−6^) were used for constraints involving ambiguous assignments or magnetically equivalent groups (e.g., methyl groups).^37^

Dihedral-angle constraints are commonly obtained from an analysis of backbone chemical shifts by programs such as TALOS+^21^ and DANGLE^27^ or, less frequently today, from experimental measurements of scalar coupling constants. As with distance constraints, Vivaldi presents these constraints and their violations interactively in the 3D viewer and as graphs. Selecting a residue with reported dihedral angle constraints will display a table as well as a Ramachandran plot and/or pie-charts for each dihedral angle as applicable, highlighting the constraint limits and angles calculated from the displayed models in a fashion similar to that shown in Supporting Information Figure S1(d).

RDC^38^,^39^ constraints are obtained from the same resource at BMRB as the distance and torsion angle constraints. Alignment tensors, which are generally not deposited together with the RDC constraints, are calculated locally by fitting one tensor per model in the NMR ensemble per alignment medium. If multiple RDC types have been deposited for a single alignment medium (e.g., N–HN, Cα–Hα, N–Cα), Vivaldi checks whether the RDC constraints were deposited as their original experimental values or as scaled values to match N–HN RDC values. The optimal alignment tensor is obtained by minimizing the RMSD between the experimental and recalculated RDC values for the alignment tensor.

The Vivaldi 3D viewer can show the principal axes of the fitted alignment tensor as three orthogonal bars tied to the molecule's orientation in the viewer and the RDC constraints are visualized by showing the atoms involved as spheres [Fig.2(e)]. Further, fitted and experimental RDC values are presented as bar and scatter plots [Fig. 2(f)]. Details about the fitted tensor, that is tensor magnitude (*D*_a_) and rhombicity (*R*), as well as three values relating to the goodness-of-fit, that is RMS deviation (Hz), Pearson *R*-value, and Cornilescu *Q*-value,^40^ are shown in a separate information section. When a residue is selected from a bar chart or in the 3D viewer, experimental and fitted values of all corresponding RDC constraints are listed.

Empirical knowledge about the structure of biological macromolecules can be used to validate local conformations and the overall fold. A number of programs, such as WHATIF,^41^ PROCHECK,^42^ WHATCHECK,^43^ and Verify3D^44^ can be used to assess the quality of macromolecular structures. More recent software packages including PSVS,^10^ ProSA-web,^45^ MolProbity,^46^ and CING^12^,^47^ combine and extend them to gain further insight into the quality of an atomistic structure model.

Residue-based quality scores are extracted from the external NRG-CING database, including a general ROG score that labels residues as red, orange, or green depending on a series of quality measures. Several PROCHECK^42^ and WHATIF^41^ scores are also shown by Vivaldi. As for any type of information, residue-based scores can be visualized as a graph, in a table or in the 3D viewer. Figure1 shows the CING ROG score for PDB entry 2k4v.^15^

For any NMR entry in the PDB, Vivaldi can be launched from the corresponding entry page at PDBe or from a direct shortcut URL (http://pdbe.org/vivaldi/NNNN where NNNN is the four-character PDB code of the entry). Alternatively, the homepage of the NMR resource at PDBe (http://pdbe.org/nmr) provides a quick-access form into which the PDB code of interest can be entered. Finally, Vivaldi can be accessed from its own webpage (http://pdbe.org/vivaldi) and the information that is displayed can be customized by means of a user-friendly wizard. On the Vivaldi page of a PDB entry, the available information can be accessed, analyzed and visualized in a multitude of ways. High-quality images from the 3D viewer and the interactive graphs can be saved for use in publications, presentations, or teaching resources. The amount and type of information that is available for a particular entry depends on what type of experimental data (if any) was deposited, whether an ensemble or a single model was deposited, and so forth. Table I lists all the types of information presented in Vivaldi, its sources, and the number of PDB entries for which it is available. All available data for each entry is assembled and presented in a consistent and intuitive way that makes it useful to both NMR experts and non-expert users.

Generating the information used by Vivaldi involves collection and format conversion of relevant data on the one hand and data presentation on the other. The first aspect is dealt with by automated Python scripts that carry out weekly checks for new, updated or removed NMR entries in the PDB and for updated validation reports. These scripts import, check, and match data in different formats and ensure their internal consistency. This is necessary as curation and annotation of coordinates and constraints is performed separately in current wwPDB curation practice. Finally, the gathered data are output as objects in JSON files, which are used for presentation to the end user. A modified version of the OpenAstexViewer 3.0 Java applet^48^ accommodating some of the specific features of Vivaldi is used as 3D display engine, while the Flotr JavaScript Plotting Library (http://solutoire.com/flotr) is used to generate charts and graphs.

## RESULTS AND DISCUSSION

Table I summarizes the types of data that can be encountered for NMR structures and the number of PDB entries for which each type of data is available in Vivaldi at the time of manuscript submission (weekly updated statistics are available from the PDBe website http://pdbe.org/nmrstats). While all NMR entries can be viewed in Vivaldi and almost all have at least a geometric validation report in the NRG-CING database, only about 60% have associated experimental constraints data deposited and processed for further analysis or visualization and only about a third have VASCO reports on chemical shift outliers at present. These numbers are set to improve, since deposition of both the experimental chemical shifts and constraints data is now mandatory (http://wwpdb.org/policy.html).

The major motivation for developing Vivaldi is to provide the users of the PDB archive (expert NMR spectroscopists and non-experts alike) with an easily accessible way to view and analyze NMR structures, their associated experimental data, and validation-related information. To demonstrate its capabilities, we present two use cases that demonstrate how Vivaldi can help evaluate the suitability of a particular entry for a user's needs.

*Example 1*. Analysis of RDCs in the context of other data for uncharacterized protein PA1076 from *Pseudomonas aeruginosa*, PDB entry 2k4v.^15^ The VASCO analysis of the chemical shifts [Fig.2(a,b)] indicates that the 23 atoms with unusual chemical shift values are spread evenly throughout the structure and many of them are in the vicinity of aromatic rings. This information confirms that the structure and chemical shift values are compatible. Furthermore, OLDERADO analysis suggests that the majority of the protein residues form one rigid body domain [Fig. 2(c), orange], including residues 91–105 (UniProt^3^ sequence numbering), which form an α-helix. However, review of the long-range distance constraints indicates that there is little support for the exact positioning of this helix [Fig. 2(d)]. Indeed, solving the structure of PA1076 with only distance and dihedral angle constraints did not allow for a reliable placement of the helix (data not shown). Introducing RDC data [Fig. 2(e,f)] allowed the positioning of helix 91–105 with more confidence.

**Figure 3:**
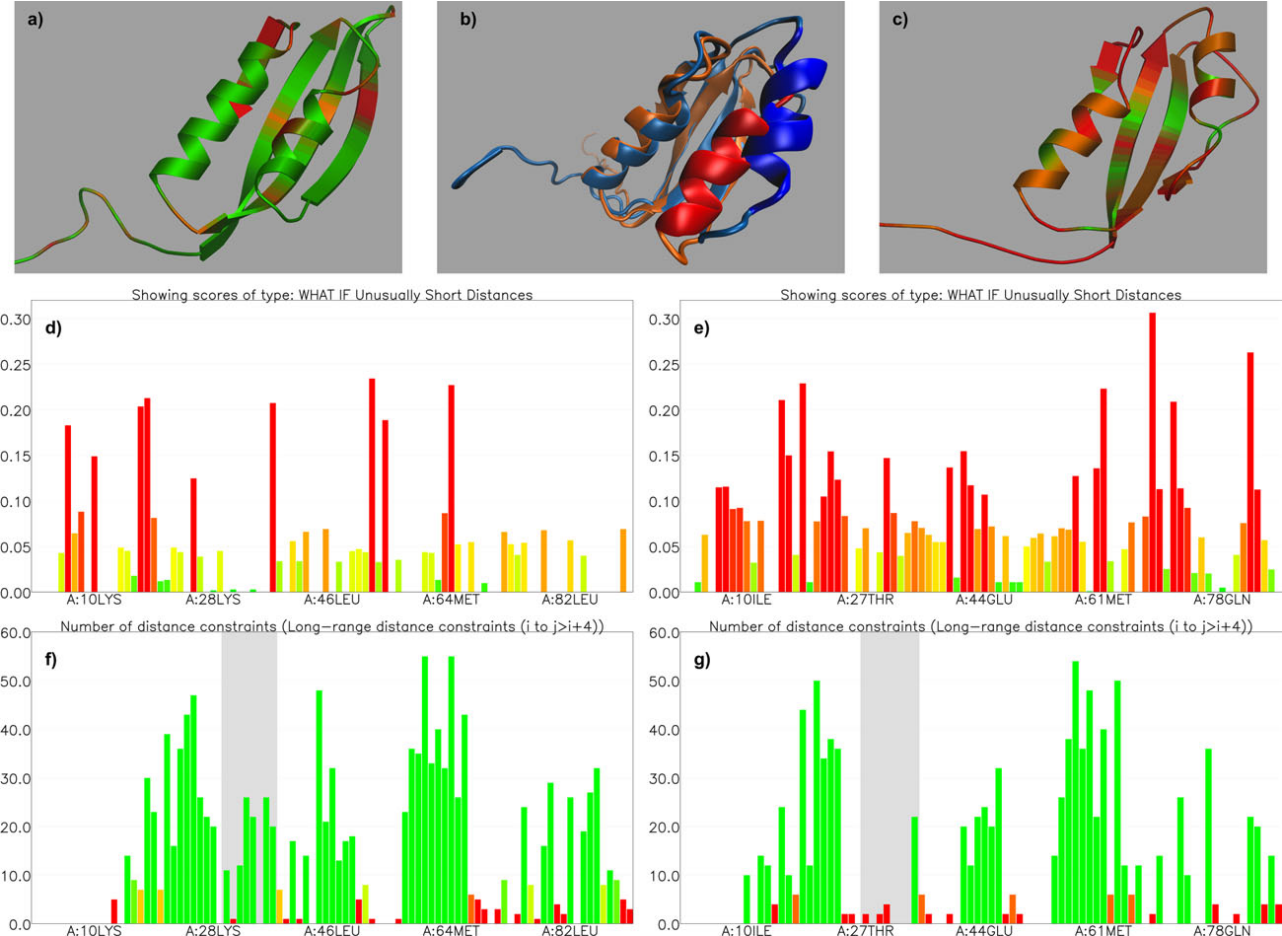
Comparison of two NMR solution structures for protein HP_0495 from *Helicobacter pylori* (PDB codes 2joq, left, and 2h9z, right). (**a**,**c**) The 3D viewers show the most representative model of each ensemble, colored by the NRG-CING red-orange-green scores, which are calculated for the entire ensemble. (**b**) Superposition of the most representative models from the two structures, highlighting the differences in the conformation of helix I and adjacent loops. Image created in VMD^54^ (**d**,**e**). Graphs of per-residue WHATIF scores reporting unusual short distances in the entire ensemble. (**f**,**g**) Graphs of the number of long-range distance constraints for each residue. Long-range constraints are defined as connecting two atoms that are five or more residues apart in the amino-acid sequence.

*Example 2*. Selecting a structure from a set of identical or similar proteins: comparing two structures for protein HP_0495 from *Helicobacter pylori* (PDB entries 2h9z^53^ and 2joq^15^), solved by different structural genomics projects. While in this case the two entries only differ by their purification tags, a similar investigation could be performed for homologous proteins. To compare two structures, one needs to launch two instances of Vivaldi in separate browser windows. In this case, the two structures are similar, but the mostly red and orange ROG scores from the NRG-CING database for entry 2h9z suggest that there may be problems with this structure [Fig.3(c)], whereas the ROG scores for entry 2joq [Fig. 3(a)] are mostly green. Comparison of several knowledge-based scores available in Vivaldi confirms that both the Ramachandran statistics (not shown) and unusually short distance analysis [Fig. 3(d,e)] are slightly less favorable for 2h9z. However, analysis of the number of long-range distance constraints available for each residue reveals that there are significant local differences between the two structures in the residue range 24–40 (UniProt^3^ sequence numbering), which spans between Strands 1 and 2 of the central β-sheet and includes helix I (residues 27–34). Only one residue from this range (Leu 34) has a significant number of long-range distance constraints (highlighted in the graph by a single green bar) to other parts of the molecule in 2h9z, while 2joq contains 10 residues that each have 10 or more long-range distance constraints (green bars), which would presumably help position helix I and surrounding loops relative to the rest of the protein with more confidence [Fig. 3(f,g)]. While a detailed analysis of the differences between the two structures is beyond the scope of this paper, use of Vivaldi reveals these differences and may direct further investigation. Indeed, overlaying the representative models from 2h9z and 2joq [Fig. 3(b)] shows that the positioning of helix I and surrounding loops differs significantly and the PDBeFold^55^ service excludes this region (residues 23–42 and 45–51) from the structural alignment of the two models. While it is not trivial to pinpoint the exact reasons for these differences, it is interesting to note that BMRB entry 15,101 associated with 2h9z has only 485 assigned chemical shift values, of which 159 belong to ^1^H nuclei, whereas BMRB entry 15,190 associated with 2joq has 1157 assigned chemical shifts, of which 653 belong to ^1^H nuclei. Completeness of assignments and consequently the number and level of ambiguity of distance constraints strongly depend on the number, types, and resolutions of collected spectra, as well as on the usually labor-intensive step of spectral analysis. At this point one can only speculate if the observed differences are due to genuine differences in experimental conditions (spectra for 2h9z were collected at 35°C, and for 2joq at 25°C), to different types and resolutions of collected data, to variations in data analysis protocols or to some combination of these factors.

## CONCLUSION

A powerful interactive web-based tool has been developed to facilitate the analysis, visualization and validation of NMR structure ensembles from the PDB. It combines structural, experimental, and validation data from a variety of sources and presents them in a consistent fashion. A user-friendly interface, including a 3D viewer, tightly coupled with charts and tables, enables both experts and non-experts in NMR structure determination to analyze NMR ensembles and to assess their quality.
