# Supplementary material for: Vivaldi: Visualization and validation of biomacromolecular NMR structures from the PDB
Source: Proteins. 2012 Nov 23;81(4):583–91. doi: 10.1002/prot.24213 (PMC3618379; doi:10.1002/prot.24213)
Supplement: Supplementary file 1 [file prot0081-0583-SD1.pdf]

Vivaldi: visualisation and validation of biomacromolecular NMR structures from the PDB (Supporting information)

Pieter M. S. Hendrickx[1], Aleksandras Gutmanas[1], Gerard J. Kleywegt[1]

[1]Protein Data Bank in Europe, European Bioinformatics Institute, Wellcome Trust Genome Campus, Hinxton, Cambridge, CB10 1SD, United Kingdom

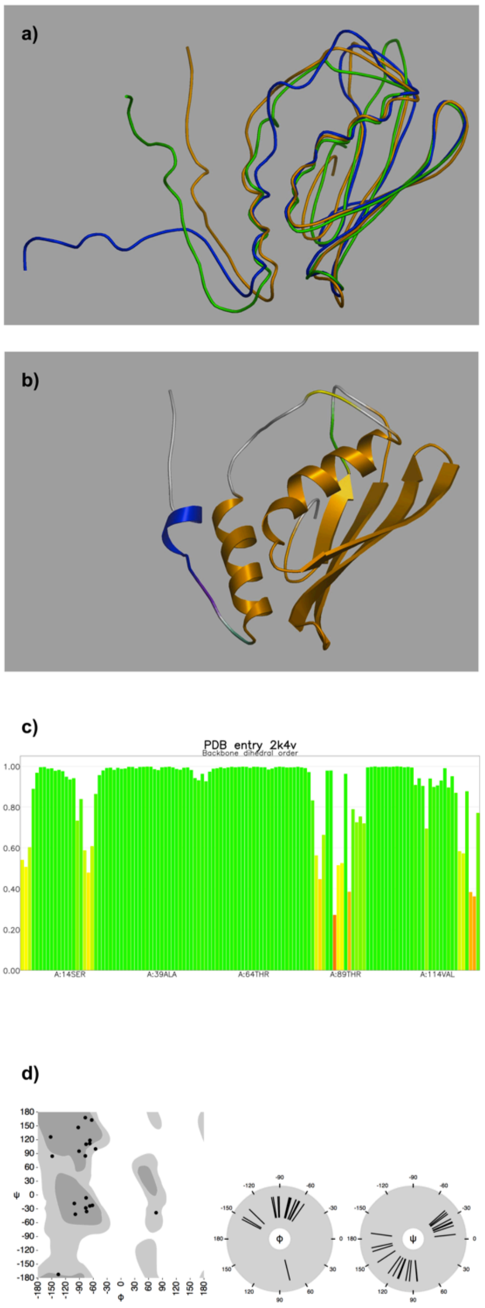

Figure S1. Various options available in Vivaldi to display conformational flexibility applied to protein PA1076 from *Pseudomonas aeruginosa* (2k4v)[15]. (a) Representative models from each cluster identified by OLDERADO[14]. (b) Ribbon view of the most representative model coloured by OLDERADO domains. (c) Dihedral order parameters per residue calculated by Vivaldi. (d) Ramachandran and pie-chart representation of backbone conformational flexibility in the deposited ensemble for residue Ile88, which is located in a poorly defined loop of the protein.
